# Leishmanicidal Activity of Aliphatic and Aromatic Lactones: Correlation Structure-Activity

**DOI:** 10.3390/molecules14072491

**Published:** 2009-07-10

**Authors:** Marcela Castaño, Wilson Cardona, Winston Quiñones, Sara Robledo, Fernando Echeverri

**Affiliations:** 1 Grupo de Química Orgánica de Productos Naturales-SIU, Universidad de Antioquia, P.O. Box 1226, Medellín, Colombia; 2 Instituto de Química, Universidad de Antioquia, Universidad de Antioquia, P.O. Box 1226, Medellín, Colombia; 3 PECET-SIU, Universidad de Antioquia, P.O. Box 1226, Medellín, Colombia

**Keywords:** metathesis, lactones, antiparasite, leishmania

## Abstract

Several aliphatic and aromatic lactones and two dimers were synthesized using the sequence: allylation - esterification - metathesis. These compounds were active *in vitro* against intracellular amastigotes of *Leishmania panamensis*. The structure-activity relationship showed the importance of the aliphatic side chain to enhance the biological activity and to obtain lower cytotoxicity. It was also observed that a decrease in the size of the lactone ring increases the selectivity index.

## 1. Introduction

Protozoan parasites are responsible for some of the most common and devastating diseases affecting humans and animals [1]. Among these diseases is leishmaniasis, which causes more deaths after malaria [2], the reason why it has become a priority for the World Health Organization (WHO) [2]. Classical antiparasite drugs are highly toxic and are largely no longer effective because of the emergence of resistance in the parasites, thus becoming a major public health problem [3]. Therefore, it is necessary to search for new active-low-toxicity molecules which may be obtained from the structural modification of natural compounds through organic synthesis.

Lactone rings are a structural feature of many natural products [4,5,6,7]. Many naturally occurring lactones, particularly α,β−unsaturated ones that are Michael acceptors, display interesting pharmacologic properties [4]. Recently, several lactones and analogues such as argentilactone [8], boronolide [9] and passifloricin A [10] have been synthesized and have exhibited high activity against *Leishmania panamensis* amastigotes.

The olefin metathesis reaction has become a powerful tool in organic synthesis. One of its most successful applications is the ring closing metathesis reaction (RCM) which affords cyclic compounds from diolefinic precursors [11,12,13,14,15,16]. Among the different kinds of cyclic compounds obtainable by RCM, unsaturated lactones of various ring sizes are achievable from α,ω−diolefinic esters using first- and/or second-generation Grubbs’s catalysts. More specifically, the preparation of α,β unsaturated γ-lactones through RCM of allyl and homoallyl acrylate have been reported using second-generation (II) Grubbs’ catalysts [17,18,19,20].

Aiming at exploring the structure-activity relationships of the products, we previously reported some structural analogues of the mentioned compounds above [10]. However, in spite of the significant leishmanicidal activity against *L. panamensis* of some of these synthetic analogues, high cytotoxicity [21] was also observed, as well as the fact that the activity does not depend on the stereochemistry of the lactone ring [10]. Based on these results other racemic lactones analogues were synthesized by introducing aromatic rings in the chain and changing the type of lactone ring. In this paper we describe the synthesis these new analogues and their activity against *Leishmania panamensis*.

## 2. Results and Discussion

### 2.1. Synthetic plan

**Figure 1:**
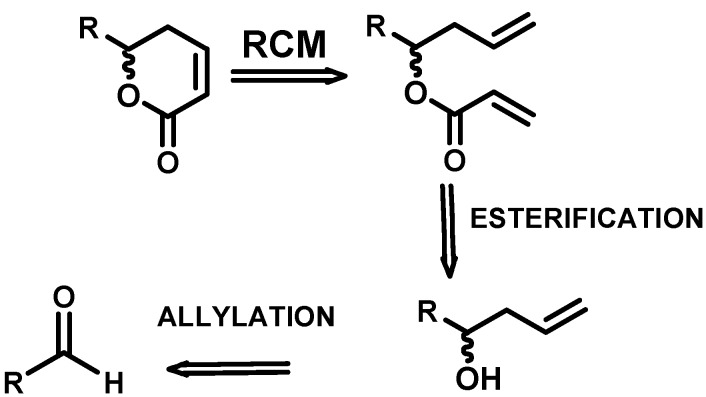
Retrosynthetic analysis of the lactone targets.

The synthesis of lactones was carried out following the methodology described in the literature [10]: allylation, esterification and ring–closing metathesis (Figure 1).

### 2.2. Synthesis of the dimer of 6-(4-hydroxy-3-methoxyphenyl)-5,6-dihydro-pyran-2-one (**7**)

**Scheme 1:**
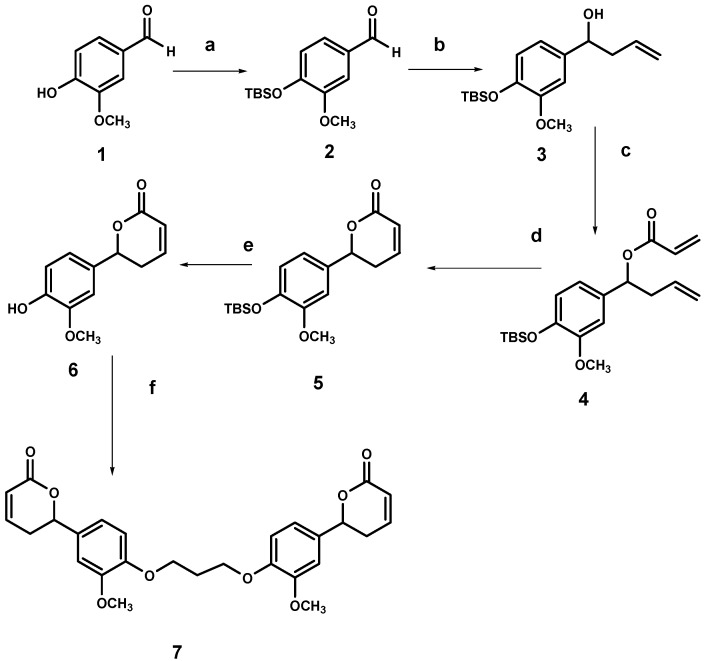
Synthesis of dimer **7**.

The synthesis of dilactone **7** is shown in Scheme 1 and discussed below. The hydroxyl group of vanillin was protected with TBSOTf [22], affording the product **2** in 99% yield. Product **2** was allowed to react with allylmagnesium bromide affording in 70% yield the homoallyl alcohol **3**, which was then treated with acryloyl chloride [23] and DIPEA to produce significant amounts of the corresponding acrylate **4**. The latter was reactive enough to undergo RCM with the first generation Grubbs ruthenium catalyst [24] PhCH=RuCl_2_(PCy_3_)_2_ with formation of the unsaturated lactone **5** in 60% yield.

*Reagents and conditions*: a) TBSTf, lutidine, CH_2_Cl_2_, 99%; b) allylmagnesium bromide, ether, 0^o^C, 70%; c) acryloyl chloride, DIPEA, CH_2_Cl_2_, 0^o^C, 97%; d) first generation Grubbs ruthenium catalyst, CH_2_Cl_2_, 60°C, 60%; e) Bu_4_NF, CH_2_Cl_2_, 90%; f) 1,3-dibromopropane, Et_3_N, acetonitrile, 69%.

Lactone **5** was allowed to react with tetrabutylammonium fluoride [25] affording lactone **6** in a 90% yield, which was then treated with 1,3-dibromopropane [26] to finally give a mixture of dilactone **7** (69%) and the monosubstitution product.

### 2.3. Synthesis of Dilactone **15**

**Scheme 2:**
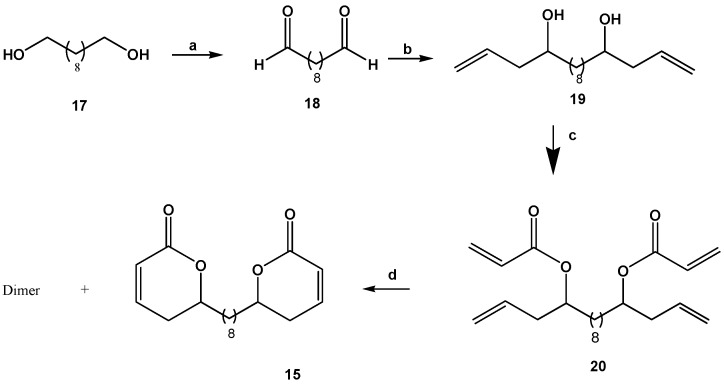
Synthesis of dilactone **15**.

The synthesis of dilactone **15** is shown in Scheme 2 and discussed below. Decanediol **17** was oxidized with PCC, giving product **18** in 65% yield. Compound **18** was allowed to react with allylmagnesium bromide affording homoallyl alcohol **19** in 43% yield. Compound **19** was treated with acryloyl chloride, DIPEA to give a 64% yield of the diester **20, **which was submitted to a metathesis reaction with the first generation Grubbs ruthenium catalyst, providing an 80:20 ratio of the dilactone **15** and the dimer in 65% yield.

*Reagents and conditions:* a) PCC, CH_2_Cl_2_, 12h, 65% b) allylmagnesium bromide, 0ºC, 3h, 43%c) Acryloyl chloride, DIPEA, CH_2_Cl_2_, 0ºC, 64% d) first generation Grubbs ruthenium catalyst, CH_2_Cl_2_, 60ºC, giving dilactone 15 and the corresponding dimer, in 65% yield (80:20 ratio).

**Figure 2:**
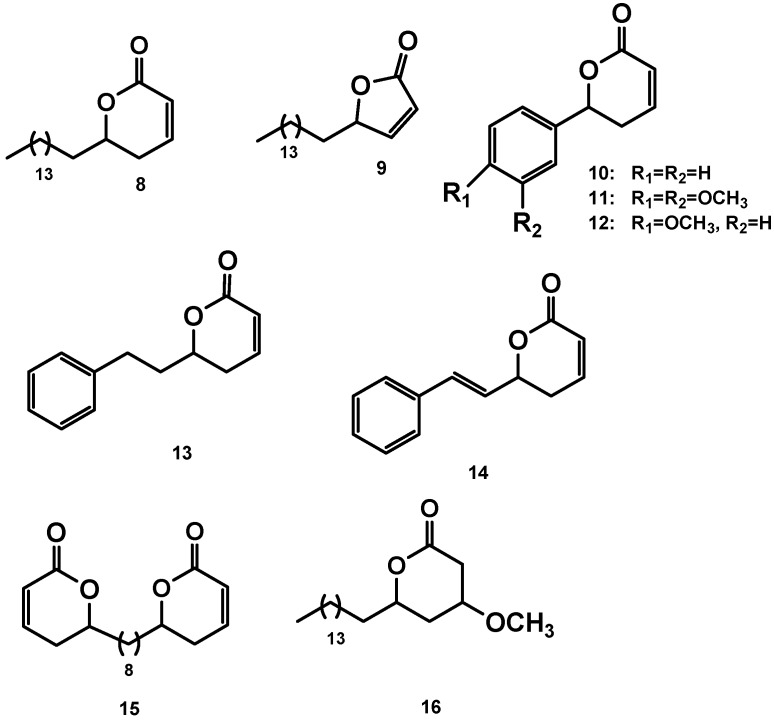
Others lactones synthesized.

Following similar synthetic strategies, we synthesized the following set of lactones (Figure 2). Lactones **13 **and **14 **have been synthesized with similar strategies and reported in the literature [27,28]

### 2.4. Synthesis of compounds **9**, **15** and the Michael adduct **16**

Lactone **9 **was prepared by treating hexadecanal with vinyl magnesium bromide, followed by the esterification and metathesis. In the same way, compound **15** was prepared following the general methodology previously described, but using decanediol as starting material [29]. Finally, compound **16** was obtained by treatment of lactone **8** with MeOH and Et_3_N. The synthesis of the remaining compounds was achieved in yields ranging 70% for the allylation (vinylation)-esterification-ring closing metathesis sequence.

### 2.5. Leishmanicidal Activity Studies

The leishmanicidal activity of synthetic compounds as well as glucantime, which was used as control drug, was evaluated following the method reported in the literature [21,30]. The results are shown in Table 1 and may provide some insights as to structure-activity relationships.

**Table 1:** Evaluation of synthetic lactones activity against *Leishmania panamensis.*

Compound	Cytotoxicity	Leishmanicidal Activity	
	LC_50_ (μg/mL)	EC_50_ (μg/mL)	SI*
**6**	27.6 ± 5.9	37.9 ± 1.4	0.7
**7**	54.1 ± 3.1	22.2 ± 3.2	2.4
**8**	3.5 ± 0.4	0.8 ± 0.2	4.4
**9**	33.9 ± 1.4	2.8 ± 0.8	12.1
**10**	1.4 ± 0.1	4.5 ± 0.3	0.3
**11**	0.4 ± 0.03	1.6 ± 0.2	0.3
**12**	1.0 ± 0.04	8.6 ± 0.3	0.1
**13**	2.5 ± 0.3	7.5 ± 2.1	0.3
**14**	2.2 ± 0.3	1.9 ± 0.1	1.2
**15**	1.0 ± 0.01	1.4 ± 0.3	0.7
**16**	51.2 ± 6.0	47.2 ±10.3	1.1
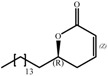			
		
	3.7^**^	0.20^**^	18.5^**^
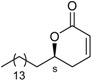			
	4.0^**^	0.22^**^	18.1^**^
		
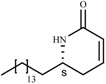			
	45.1^**^	3.42^**^	13.2^**^
		
		
**Glucantime**	416.4	6.7	59.6

*SI=LC_50_/EC_50_; ** biological results reported in the literature [10].

Not only the presence of an α,β-unsaturated lactone, but also a less polar portion which can be a long aliphatic chain (compound **8**) or an aromatic ring (compounds **10,14**) appears essential for leishmanicidal activity. The change of the aliphatic side chain (compound **8**) for an aromatic ring (compounds **10-14**) led to an increase in cytotoxicity and a decrease in activity; however, this ring should not be directly attached to the lactone (**6**, **13** vs. **14**). Additionally, functionalization causes change in biological activity, which is affected by the relative position in the aromatic ring (**10** vs **11** and **12**), although cytotoxicity is high still. On the other hand, a large molecule such as dimer **7** is less active, even though it has two lactone rings, probably due to steric hindrance of the interaction with a putative receptor, but dimer **15, **which also has two lactone rings, displays a significant activity, even though it is smaller than compound **7. **However, dimer **15 **has a similar activity to compound **8, **but improved cytotoxicity. Perhaps, the improved activity of **15 **is related to the lipophilic central chain joining the lactone rings. However, the central chain cannot be as large as in the case of dimeric molecule **7, **otherwise activity will be compromised.

Regarding changes in the lactone ring, the reduction of the ring size (compound **9**) leads to a considerable decrease in cytotoxicity (3.5 vs 33.9, **8** vs **9**), but its activity is three times lower (2.8 vs 0.8 compound **8**). However, surprisingly, the γ-lactone shows a high level of leishmanicidal activity and its cytotoxicity is lower than for the δ-lactone. In Michael adduct **16** both cytotoxicity and activity decrease significantly, which confirms the importance of the α,β-unsaturated carbonyl system in this type of compounds.

In general, most of the compounds have significant activity in *L. panamensis* amastigotes, exhibiting an EC_50_ of less than 10.0 mg/mL (Table 1). With regard to selectivity index (SI = LC_50_/EC_50_) compound **9** (SI = 12.1) would be the most promising, because of its simple structure and low LC_50_ (33.9). These results are comparable with the previously reported leishmanicidal activity of lactams [7].

## 3. Experimental

### 3.1. General

NMR spectra were recorded as CDCl_3_ solutions on a Bruker AMX 300 instrument operating at 300 MHz for ^1^H and 75 MHz for ^13^C. Chemical shifts (δ) are expressed in ppm with the solvent peak as reference; coupling constants (*J*) are given in Hertz (Hz). Multiplicity assignments of ^13^C signals were made by means of the DEPT pulse sequence. High resolution mass spectra were run in the electron impact mode (EIMS 70 eV). IR data were measured with oily films on KBr pellets (solids) on a Perkin-Elmer RXI-2000 (FT-IR). Experiments which required an inert atmosphere were carried out under dry N_2_ in flame-dried glassware. Et_2_O and THF were freshly distilled from sodium/benzophenone and transferred via syringe. Dichloromethane was freshly distilled from CaH_2_. Commercially available reagents were used as received. Unless otherwise indicated, "work-up" means pouring the reaction mixture into brine, followed by extraction with the solvent indicated in parenthesis. If the reaction medium was acidic (basic), an additional wash with 5% aq NaHCO_3_ (aq NH_4_Cl) was performed. Washing with brine, drying over anhydrous Na_2_SO_4_ and elimination of the solvent under reduced pressure were followed by chromatography on a silica gel column (60-200 µm) with the indicated eluent.

### 3.2. General synthetic procedures

*4-(tert-Butyldimethylsilanyloxy)-3-methoxybenzaldehyde* (**2**): Vanillin (**1, **1 g, 6.57 mmol) was dissolved under N_2 _in dry CH_2_Cl_2 _(20 mL) and treated sequentially with 2,6-lutidine (1.14 mL, 9.86 mmol) and TBSOTf (1.66 mL, 7.23 mmol).The reaction mixture was then stirred for 1 h at 0ºC. After this time the reaction mixture was poured onto a saturated aq. NH_4_Cl solution and extracted with CH_2_Cl_2_. Column chromatography on silica gel (hexanes-EtOAc 4:1) afforded the desired silylated derivative **2 **in 99% yield. ^1^H-NMR: δ 0.00 (s, 6H), 0.85 (s, 9H), 3.62 (s, 3H), 6.61 (dd, 2H, *J* = 9.3 Hz, *J* = 8 Hz), 6.70 (s, 1H), 9.80 (s, 1H);^ 13^C-NMR: δ 18.30 (C), 25.50 (CH_3_), 46.80 (CH_3_), 73.60 (CH), 110.20 (CH), 118.10 (CH), 120.20 (CH), 137.30 (C), 143.60 (C), 150.30 (C), 189.00 (CH).

*1-[4-(tert-Butyldimethylsilanyloxy)-3-methoxyphenyl]-but-3-en-1-ol* (**3**): The aldehyde **2** (0.18 mmol) was dissolved in dry ether (50 mL) a 0ºC, allylmagnesium bromide (0.18 mmol, 1M solution in THF) was added and then the mixture was stirred for 4 h under N_2_. The reaction mixture was then quenched through addition of a saturated aq. solution of NH_4_Cl and extracted with Et2O. The combined organic phases were washed with a saturated aq. solution of NaCl, and dried with Na_2_SO_4. _After filtration and evaporation of the solvent under vacuum the residue was column chromatography on silica gel (hexanes-EtOAc 8:2 and then 3:2) to afford the homoallylic alcohol in 70% yield. ^1^H-NMR: δ 0.00 (s, 6H), 0.85 (s, 9H), 2.29 (t, 2H, *J* = 6.8 Hz), 3.62 (s, 3H), 4.43 (t, 1H, *J* = 6.5 Hz), 4.90-4.96 (m, 1H), 5.57-5.63 (m, 2H), 6.61 (dd, 2H, *J* = 9.3 Hz, *J* = 8 Hz), 6.70 (s, 1H); ^13^C-NMR: δ 18.32, 25.52 (CH_3_), 43.84 (CH_2_), 46.86 (CH_3_), 73.65 (CH), 110.20 (CH), 117.70 (CH_2_), 118.11 (CH), 120.22 (CH), 134.83 (CH), 137.34 (C), 150.38 (C), 143.66 (C).

*Acrylic acid 1-[4-(tert-butyldimethylsilanyloxy)-3-methoxyphenyl]-but-3-enyl ester* (**4**): Alcohol **3** (0.75 mmol) was dissolved under N_2_ in dry CH_2_Cl_2_ (11 mL), cooled to 0°C, and treated sequentially with DIPEA (3.74 mmol) and acryloyl chloride (32.26 mmol). The reaction mixture was stirred for 2 h at 0°C and then work-up (extraction with CH_2_Cl_2_). Column chromatography on silica gel (hexanes-EtOAc 4:2) afforded ester **4 **in 97% yield. ^1^H-NMR: δ 0.14 (s, 6H), 0.99 (s, 9H), 2.50-2.70 (m, 2H), 3.80 (s, 3H), 5.01-5.11 (m, 3H), 5.79- 5.85 (m, 2H), 6.10-6.20 (m, 1H), 6.41 (dd, 1H, *J* = 1.0 Hz, *J* = 17.3 Hz), 6.70-6.80 (m, 2H), 6.80 (s br, 1H); ^13^C-NMR: δ 18.30 (C), 26.49 (CH_3_), 40.39 (CH_2_), 55.48 (CH_3_), 75.19 (CH), 110.71(CH), 117.99 (CH), 119.00 (CH_2_), 120.50 (CH), 128.60 (CH), 130.80 (CH_2_), 133.30 (CH), 134.50 (C), 144.70 (C), 150.70 (C), 165.34 (C).

*6-[4-(tert-Butyldimethylsilanyloxy)-3-methoxyphenyl]-5,6-dihydropyran-2-one* (**5**)**: **Compound **4 **(0.86 mmol) was dissolved under N_2_ in dry, degassed CH_2_Cl_2_ (35 mL) and treated with ruthenium catalyst PhCH=RuCl_2_(PCy_3_)_2_ (10% mmol). The mixture was heated at reflux until consumption of the starting material (ca. 3 h, TLC monitoring!). Solvent removal in vacuum and column chromatography on silica gel (hexanes-EtOAc, 9:1) furnished lactone **5** in 60% yield. ^1^H-NMR: δ 0.00 (s, 6H), 0.84 (s br, 9H), 2.37-2.57 (m, 2H), 3.67 (s, 3H), 5.22 (dd, 1H, *J*=4.3Hz, *J*=7.4Hz), 5.96 (d, 1H, *J*=9.8Hz), 6.63-6.70 (m, 2H), 6.78- 6.83 (m, 2H); ^13^C-NMR: δ 18.18, 25.29(CH_3_), 31.60(CH_2_), 55.45(CH_3_), 79.32(CH), 109.94(CH), 118.60(CH), 120.64(CH), 121.48(CH), 131.85 (C), 144.89(CH), 145.31 (C), 151.13 (C), 164.44 (C).

*6-(4-Hydroxy-3-methoxyphenyl)-5,6-dihydropyran-2-one* (**6**): The silylated lactone **5** (0.1 g, 0.3 mmol) was dissolved in THF (10 mL), treated with TBAF (5 mg, 0.0007 mmol) and the mixture stirred for 9 h at 25ºC. The reaction was then quenched by addition of a solution of NH_4_Cl, and after work-up (CH_2_Cl_2_), the organic phase was washed with a saturated NaCl solution, dried with Na_2_SO_4, _and evaporated under vacuum. The residue was subjected to column chromatography on silica gel (hexanes-EtOAc 1:1 and 3:2) furnishing lactone **6 **as an amorphous solid in 89.3 % yield. Mp: 109-111 ºC; IR (KBr) ν_max_ (cm^-1^): 3392 (OH), 1709 (C = O), 1136 (C(= O)-O), 1033 (O – C = C, O-CH_3_)**; **^1^H-NMR: δ 2.51- 2.69 (m, 2H), 3.91 (s, 3H), 5.38 (dd, 1H, *J*=4.5Hz, *J*=11.5Hz), 5.72 (s br, 1H), 6.13 (dd, 1H, *J*=2.2Hz, *J*=9.8Hz), 6.85- 6.90 (m, 2H), 6.90 -7.00 (m, 2H); ^13^C-NMR: δ 32.02(CH_2_), 56.16(CH_3_), 79.62(CH), 109.03(CH), 114.50(CH), 119.50(CH), 121.93(CH), 130.88, 145.21(CH), 146.69, 147.48; HR EIMS, m/z 221.1902 (M+H).

*Dimer of 6-(4-hydroxy-3-methoxyphenyl)-5,6-dihydropyran-2-one* (**7**)*:* Lactone **6** was dissolved in acetonitrile (10 mL), Et_3_N (1.5 eq, 2.3 mL) was added and the reaction mixture stirred for 15 min. Then 1,3-dibromopropane (0.5 eq, 0.02 mL) was added, and the resulting mixture was heated at reflux for 8 h. After this time an aq. solution of HCl was added and the mixture was extracted with EtOAc. The combined organic phases were dried over Na_2_SO_4_, filtered and concentrated under vacuum. The residue was subjected to column chromatography on silica gel (hexanes-EtOAc 9:1) to furnish dimeric compound **7 **in 69 % yield. Mp: 81-83ºC; I.R (KBr) ν_max_ (cm^−1^): 1708 (C = O), 1171 (C(= O)-O), 1128 (O – C = C, O-CH_3_); ^1^H-NMR: δ 2.21- 2.26 (m, 2H), 2.32-2.41 (m, 2H), 3.89 (s, 3H), 4.15-4.32 (m, 4H), 5.28-5.44 (m, 2H), 5.65-5.97 (m, 2H), 6.78-7.01(m, 8H); ^13^C-NMR: δ 29.69 (CH_2_), 56.061 (CH_3_), 61.89 (CH_2_), 109.82 (CH), 123.659 (CH), 133.220, 145.97 (CH); HR EIMS, (M+H): 481.2

*Dilactone 6,6'-octane-1,8-diylbis(5,6-dihydro-2H-pyran-2-one)* (**15**)*:* Amorphous solid. Mp: 81-84 ºC; IR (KBr) ν_max_ (cm^-1^): 1698 (C = O), 1262 (C(= O)-O), 1028 (O – C = C); ^1^H-NMR: δ 1.25-1.37 (m, 14H), 1.56-1.67 (m, 4H), 2.30-2.35 (m, 4H), 4.37-4.46 (m, 2H), 6.02 (d, *J* = 9.8 Hz, 2H_2_), 6.87 (m, 2H_3_); ^13^C-NMR: δ (CH_2_), 29.90-30.10 (6CH_2_), 35.50 (CH_2_), 78.20 (CH), 122.10 (CH), 145.80 (CH), 165.20; HR EIMS: (M+H): 307.1911 (C_18_H_27_O_4_), calc. 307.1909.

*6-Dodecyl-4-methoxytetrahydro-pyran-2-one* (**16**): The lactone **8** (200 mg, 0.962 mmol) was dissolved in MeOH (30 mL), and treated with Et_3_N (0.962 mmol). The mixture was heated at reflux until the starting material was consumed (ca. 4 h, TLC monitoring); after this time, the mixture is neutralized with a solution of sodium bicarbonate, extracted with ethyl acetate, and the organic phase dried with Na_2_SO_4_, concentrated and purified by column chromatography (silica gel, hexane: ethyl acetate 1:1 ) to give an amorphous solid. Mp: 54-56 ºC; IR (KBr) ν_max_ (cm^-1^): 1724 (C = O), 1245 (C(= O)-O), 1081 (O – C = C, O-CH_3_); ^1^H-NMR: δ 0.88 (t, 3H, *J*=6.6Hz), 1.25-1.38 (m, 26 H), 1.60-1.70 (m, 2H), 2.03-2.17 (m, 2H), 2.67-2.69 (m, 2H), 3.33 (s, 3H), 3.70-3.80 (m, 1H), 4.49-4.58 (m, 1H)**; **^13^C-NMR: δ 14.11 (CH_3_), 22.68 - 29.69 (10CH_2_), 31.92 (CH_2_), 33.17 (CH_2), _35.52 (CH_2_), 56.09 (CH_3_), 71.47(CH), 75.91 (CH); HR EIMS, (M+H): 341.3044 C_21_H_41_O_3_ calc.341.3056.

### 3.3. Bioassays

Leishmanicidal activity and cytotoxicity were measured according to previously reported procedures [21].

## 4. Conclusions

A number of aliphatic and aromatic lactones as well as two dimers were synthesized and evaluated against amastigotes of *Leishmania panamensis*. The analysis of the results showed that the lactones with an aliphatic side chain are more active and less cytotoxic than lactones with aromatic side chains. We also found that reducing the size of the lactone ring results in increased activity and selectivity index. Furthermore, it confirms the importance of the α,β-unsaturated lactone moiety for biological activity in this area.
